# Identification of Mannose‐Capped‐Arabinomannan 101‐mer as a Potential Influenza Virus Vaccine Adjuvant

**DOI:** 10.1002/advs.202519843

**Published:** 2026-02-03

**Authors:** Yu‐Fang Zhang, Ruihong Xu, Jia Luo, Yuxin Ma, Guozhi Xiao, Ye Liu

**Affiliations:** ^1^ Institute of Medical Biology Chinese Academy of Medical Sciences and Peking Union Medical College Kunming Yunnan P. R. China; ^2^ Kunming Medical University Kunming Yunnan P. R. China; ^3^ State Key Laboratory of Phytochemistry and Natural Medicines Kunming Institute of Botany University of Chinese Academy of Sciences Chinese Academy of Sciences Kunming Yunnan P. R. China; ^4^ State Key Laboratory of Respiratory Health and Multimorbidity Beijing P. R. China; ^5^ Key Laboratory of Pathogen Infection Prevention and Control (Peking Union Medical College) Ministry of Education Beijing P. R. China

**Keywords:** adjuvant, immunogenicity, influenza vaccinations, mannose‐capped arabinomannan

## Abstract

Many natural bacterial components as adjuvants can activate the host immune system, but the excessive toxicity and structure‐identification challenge limit their applications and structure‐activity relationship studies. Herein, we report the role of a series of chemically synthesized mannose‐capped arabinomannan motifs from *Mycobacterium tuberculosis* cell wall, including 18‐mer, 19‐mer, 27‐mer, and 101‐mer in regulating host immunity. As an influenza vaccine adjuvant, 101‐mer induced significantly enhanced anti‐influenza antibody response and immune protection compared with other arabinomannan motifs. 101‐mer elicited robust immunoenhancement while exhibiting a favorable tolerability profile, as it did not trigger any observable physiological toxicity or inflammatory reactions in various organs. Mechanistically, we found 101‐mer may serve as a Dectin‐2 agonist to activate host immunity through Syk/NF‐κB signaling. This study provided a new oligosaccharide candidate that can satisfy the required “efficacy‐safety” balance for clinical adjuvant development.

## Graphical abstract

Well‐defined and synthetic mannose‐capped arabinomannan 101‐mer from *Mycobacterium tuberculosis* cell wall was identified as a potent influenza vaccine adjuvant, boosting the antibody response, realizing full protection and showing excellent safety.

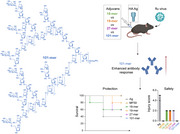

Adjuvants play a critical role in enhancing vaccine efficacy [1, 2, 3]. Among them, bacterial components such as oligosaccharides and glycoconjugates have attracted increasing attention due to their potent immunostimulatory activity [4, 5]. However, many bacterial‐derived immunostimulatory components are associated with severe systemic toxicity, thereby largely preventing their clinical translation. In general, such components are isolated from natural sources, which have a high structural heterogeneity and are very difficult to precisely define their chemical structures [6, 7]. Encouragingly, the recent achievements in the fully synthetic field enable the researchers to artificially produce bacterial oligosaccharides with the precise structural definition and tunable immunostimulatory properties [8, 9, 10, 11, 12, 13, 14, 15, 16, 17, 18, 19, 20, 21, 22, 23]. It hence provides a chance to illuminate the structure‐activity relationship, finally realizing an efficacy‐safety balance for adjuvant development.

Influenza virus remains a major global health threat, causing an estimated 1 billion infection cases and up to 650 000 deaths globally each year [24, 25]. Vaccination is one of the most effective strategies to reduce influenza‐related morbidity and mortality. Incorporation of adjuvants can enhance vaccine efficacy, yet most licensed influenza adjuvants—such as MF59 and AS03—are complex, multicomponent formulations [26, 27]. Their compositional complexity results in complicated mechanistic dissection and rational optimization. Moreover, aluminum‐based adjuvants, though widely applied, fail to elicit strong cellular immune responses [28]. Therefore, developing advanced adjuvants to improve influenza vaccine efficacy became an emergency.

To overcome this challenge, we here used the well‐defined and synthetic oligosaccharides from *Mycobacterium tuberculosis* cell walls as the influenza vaccine adjuvant. We evaluated the structure‐activity relationships and immunoregulatory mechanisms of a series of mycobacterial mannose‐capped arabinomannan motifs, including 18‐mer, 19‐mer, 27‐mer, and 101‐mer. Notably, we identified a fully synthetic arabinomannan 101‐mer as a single‐component adjuvant, which markedly enhances influenza vaccine immunogenicity and protection.

Recently, we applied an orthogonal one‐pot glycosylation strategy on the basis of glycosyl *ortho*‐(1‐phenylvinyl benzoates) [29] to achieve modular synthesis of a series of well‐defined arabinomannan motifs, including the 18‐mer, 19‐mer, 27‐mer, and 101‐mer from *Mycobacterial tuberculosis* cell wall (Figures 1; S1–S8) [7], which were evaluated as potential adjuvants.

**FIGURE 1:**
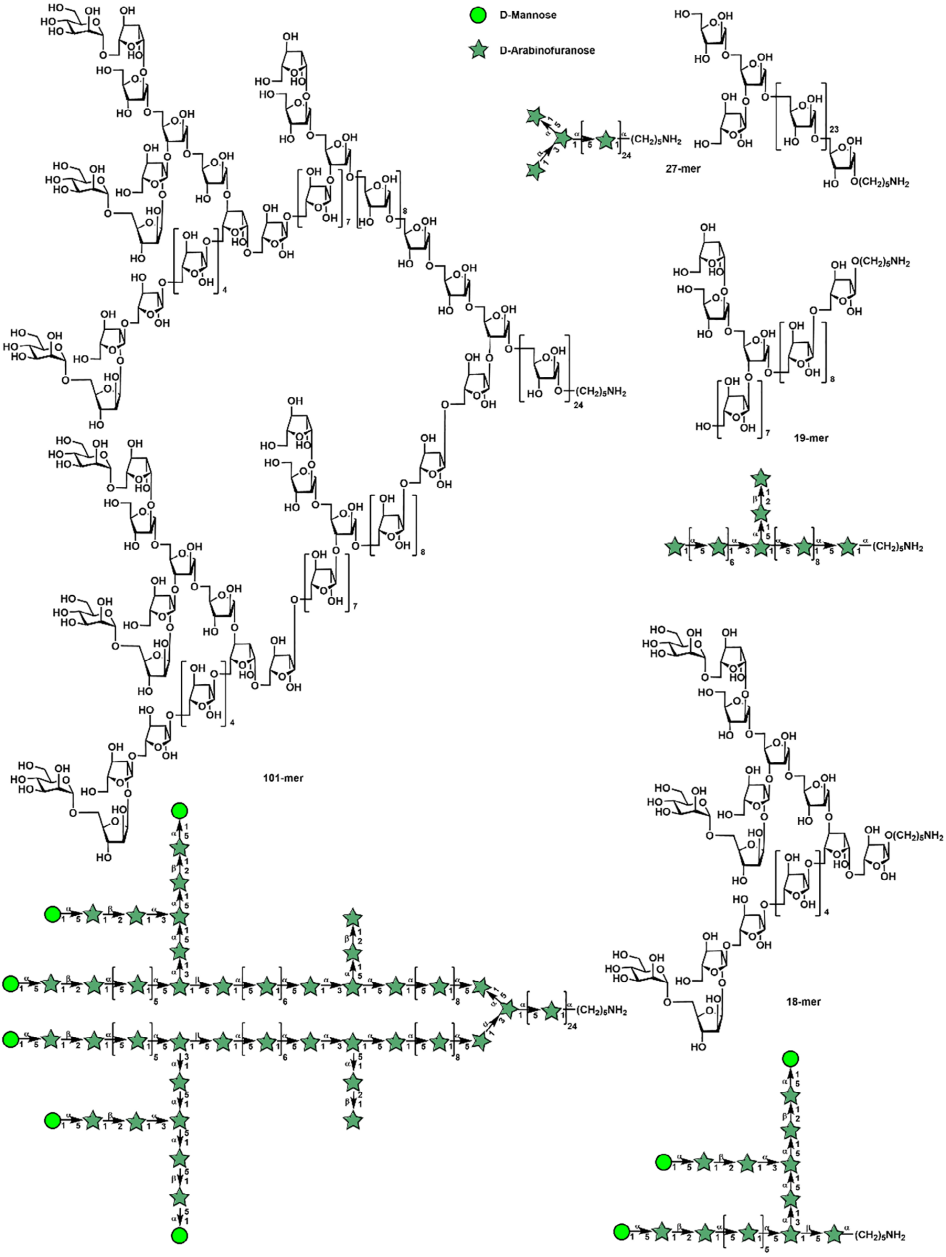
Chemical structures of arabinomannan motifs (18‐mer, 19‐mer, 27‐mer, and 101‐mer) from *Mycobacterial tuberculosis* cell wall.

Dendritic cells (DCs), as key antigen‐presenting cells (APCs) [30, 31], were used to assess the adjuvant performance of our synthetic arabinomannan motifs in vitro (Figure S9). Among the four structurally defined motifs (18‐mer, 19‐mer, 27‐mer, and 101‐mer), 101‐mer, rather than other motifs (18‐mer, 19‐mer, and 27‐mer), markedly boosted DC phagocytosis. Notably, the phagocytic activity induced by the 101‐mer exceeded that observed with MF59, a clinically used influenza vaccine adjuvant. This result showed the strong activity of 101‐mer in promoting antigen presentation.

Encouraged by the activity of 101‐mer in facilitating DC phagocytosis, we next evaluated the in vivo performance of synthetic arabinomannan motifs in improving influenza vaccine efficacy (Figure 2a).

**FIGURE 2:**
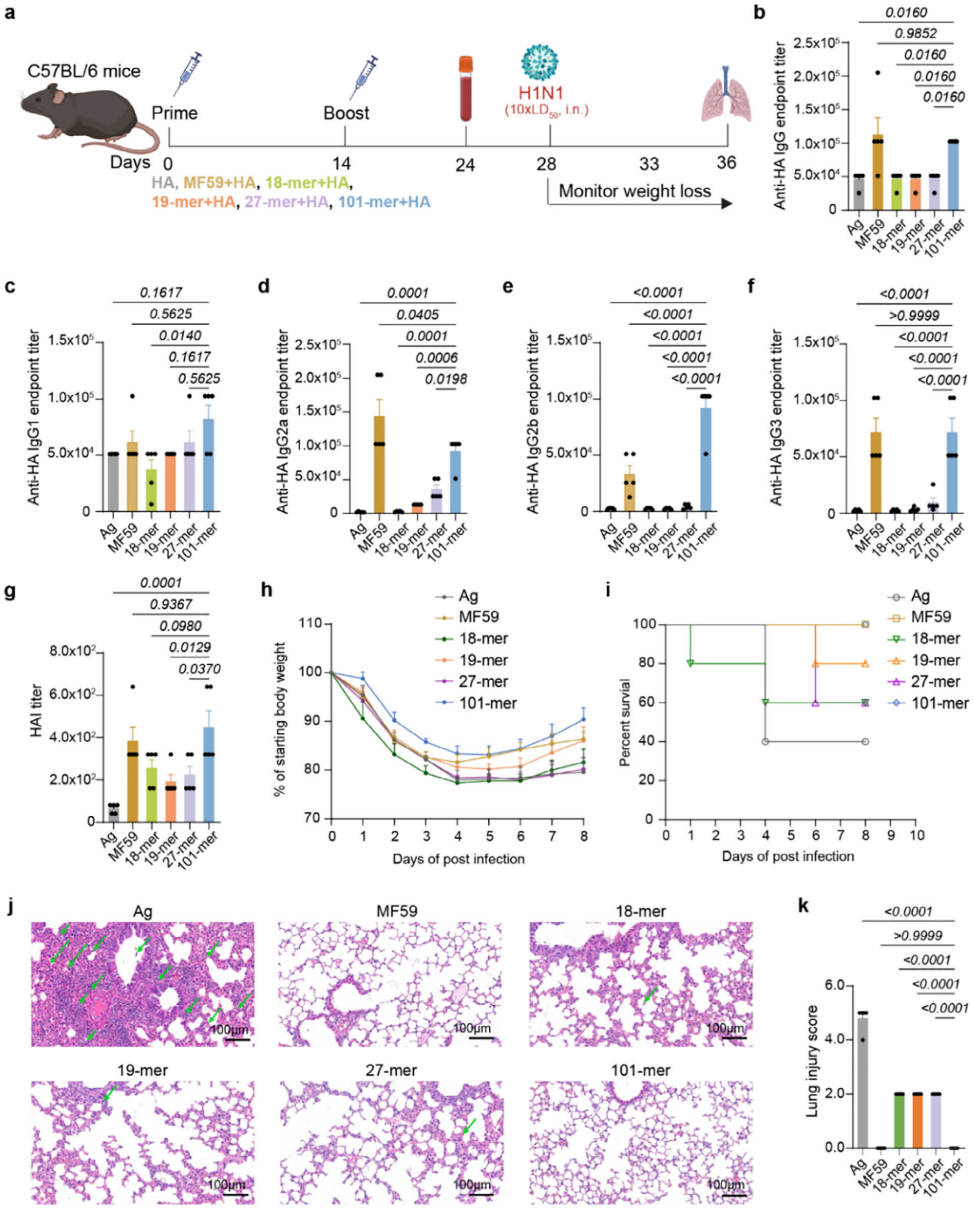
101‐mer safely and potently enhances influenza vaccine immunity. (a) Immunization and influenza infection regimen. C57BL/6 mice (6‐8 weeks old) were immunized twice (weeks 0 and 2) with different vaccine formulations: Ag group (10 µg HA per each injection), MF59 group (10 µg HA + 50 µL MF59 per each injection), 18‐mer group (10 µg HA + 10 µg 18‐mer per each injection), 19‐mer group (10 µg HA + 10 µg 19‐mer per each injection), 27‐mer group (10 µg HA + 10 µg 27‐mer per each injection) and 101‐mer group (10 µg HA + 10 µg 101‐mer per each injection). On day 24, serum samples were collected for analysis. On day 28, mice were challenged intranasally (i.n.) with 10xLD_50_ of H1N1. Body weights were recorded for 8 days. On day 8 post‐infection, mice were sacrificed, and lungs were collected for histological analysis. (b–f) HA‐specific antibody titers determined by ELISA: total IgG (b), IgG1 (c), IgG2a (d), IgG2b (e), and IgG3 (f). (g) Hemagglutination inhibition (HAI) titers. (h) Weight loss curve after H1N1 infection. (i) Survival curve after H1N1 infection. Mice with a body weight loss of more than 25% were humanely euthanized. (j–k) H&E staining of pulmonary pathological lesions (j) with corresponding lung injury scoring (k) on day 8 post‐infection. Green arrows indicate the presence of inflammatory cell infiltrates. Scale bars, 100 µm. All data were shown as mean ± s.e.m. Statistical significance was tested with one‐way ANOVA. *n* = 5 mice per group.

Influenza poses a global health threat [24, 25]. Inducing a potent antibody protection is critical for controlling the epidemic spread [32]. Our data showed that 101‐mer significantly enhanced influenza antigen hemagglutinin (HA)‐specific antibody response, reaching a magnitude comparable to MF59, but much higher than other shorter motifs (Figure 2b). Antibody subtype responses further showed that 101‐mer broadly amplified vaccine‐induced IgG1, IgG2a, IgG2b, and IgG3 responses (Figure 2c–f), suggesting an amplification of antiviral activity [33]. Functional antibody activity assessment, based on hemagglutination inhibition (HAI), further confirmed that 101‐mer induced antiviral activity comparable to MF59 and superior to shorter motifs (Figure 2g).

We next carried out a viral challenge to investigate arabinomannan‐adjuvanted protective efficacy. 101‐mer–adjuvanted vaccination provided complete protection from H1N1 infection, achieving a level of protection comparable to MF59. Among all mouse groups, 101‐mer‐treated mice obtained minimal weight loss and faster weight recovery than all other groups (Figure 2h). Moreover, the 101‐mer group reached a 100% survival rate, similar to MF59, and was significantly higher than that in other shorted motifs groups (60% for 18‐mer, 80% for 19‐mer, and 60% for 27‐mer, Figure 2i). Influenza virus infection would lead to lung injury [24, 34]. Lung pathology becomes a key tool to indicate influenza virus‐caused severity. 101‐mer‐adjuvanted vaccination markedly reduced the inflammatory infiltration and lung tissue damage in challenge mice, comparable to MF59, whereas mice from HA alone, and shorter motifs still exhibited moderate to severe lung damage (Figure 2j,k). Taken together, 101‐mer, as an influenza adjuvant, showed significantly better immunoregulatory activity and protective capacity in vivo.

To investigate the immunoregulatory mechanisms of 101‐mer adjuvant, we first performed bulk RNA sequencing from mouse spleens. Transcriptome analysis identified 169 differentially expressed genes (DEGs, absolute log2 fold change > 1, *P*_adj_ < 0.05) using Deseq2 [35], with distinct clustering from controls (Figure S10a–c). Furthermore, GO enrichment [36, 37] showed significant upregulation of immune‐related pathways, including immunoglobulin‐mediated immune response, B cell‐mediated immunity, and antigen binding (Figure 3a). Via network analysis (WGCNA) [38], the critical role of the Dectin‐2 signaling pathway was highlighted (Figure 3b). Dectin‐2, a C‐type lectin receptor recognizing mannose‐rich glycans, is well‐known to initiate Syk/NF‐κB signaling and promote type I interferon production to activate the host innate immune system [39, 40]. This analysis supported that 101‐mer adjuvant can improve influenza vaccine efficacy through Dectin‐2‐mediated immunoactivation.

**FIGURE 3:**
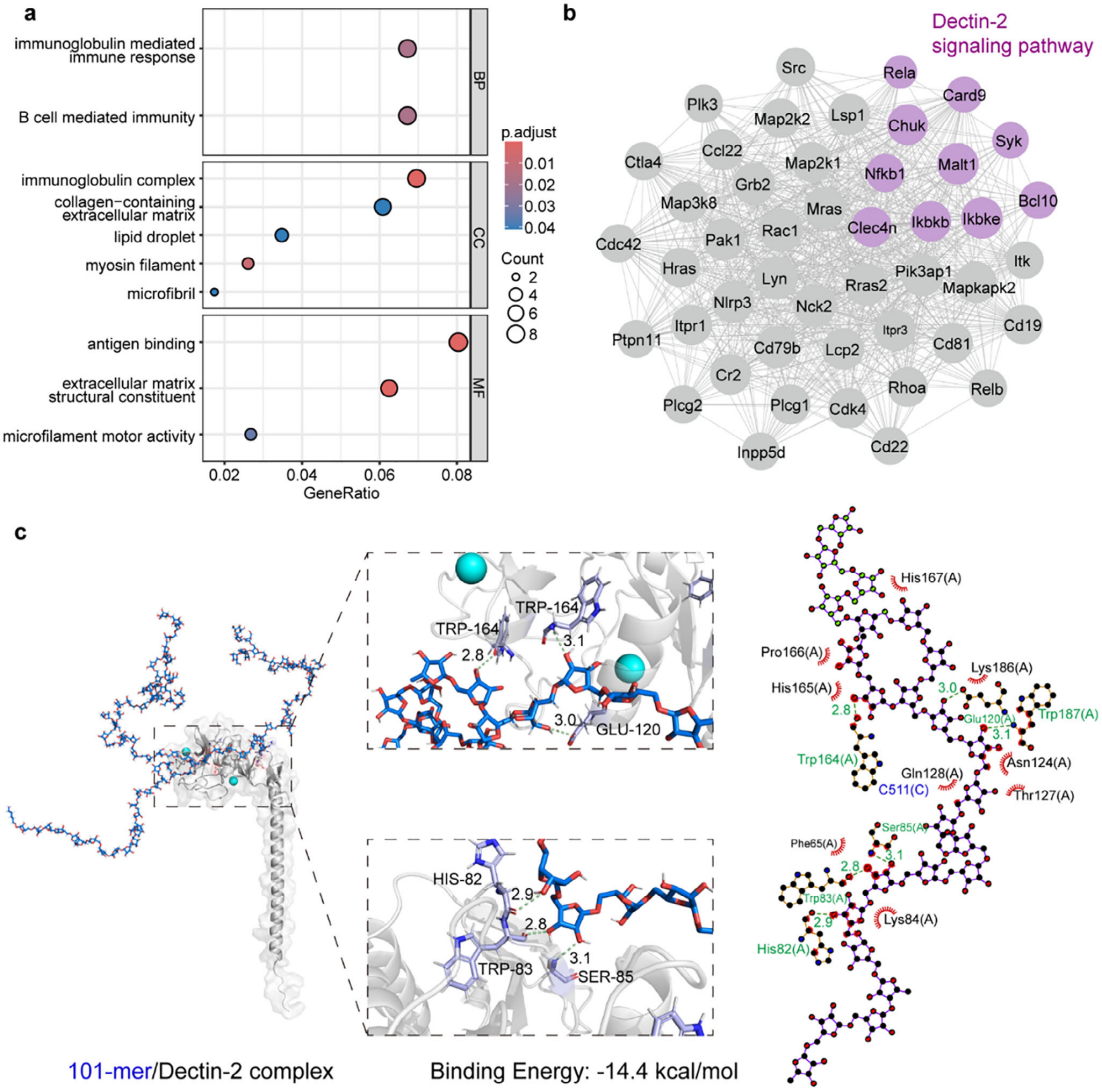
101‐mer is associated with putative Dectin‐2–related immunoregulatory effects. (a) Bulk RNA‐seq of spleens from 101‐mer–treated mice revealed 169 differentially expressed genes enriched in immune‐related pathways, including immunoglobulin‐mediated immune response, B cell‐mediated immunity, and antigen binding. (b) Co‐expression gene network of DEGs, especially the Dectin‐2 signaling pathway‐related gene co‐expression network. (c) Molecular docking of 101‐mer (blue) with the Dectin‐2 CRD (gray) revealed the strongest binding (−14.4 kcal/mol), stabilized by six hydrogen bonds (green; HIS82, TRP83, SER85, TRP164, GLU120, TRP187).

To further investigate the possible structural basis, molecular docking was performed between four arabinomannan motifs and murine Dectin‐2 [41]. Docking analysis showed that all motifs bound the conserved calcium‐dependent glycan‐binding pocket with distinct affinities. The 101‐mer exhibited the strongest binding (−14.4 kcal/mol), stabilized by six hydrogen bonds (HIS82, TRP83, SER85, TRP164, GLU120, TRP187; Figure 3c). In contrast, 18‐mer and 19‐mer showed moderate binding (−11.7 and −12.0 kcal/mol; Figure S11a,b), and 27‐mer showed the weakest interaction (−11.1 kcal/mol; Figure S11c). Together with downstream signaling readouts, these results suggest a putative role for Dectin‐2–dependent Syk/NF‐κB signaling. However, the precise mode of receptor engagement remains unclear, and docking provides only a hypothesis‐generating framework. Further experimental validation is required to confirm this putative mechanism.

The in vivo tolerability of the arabinomannan motifs was systematically evaluated. In our study, 101‐mer did not induce observable adverse effects on the loss of body weight (Figure 4a), organ coefficient (Figure 4b), or abnormal changes in serum biochemical markers (aspartate aminotransferase/AST, alanine aminotransferase/ALT, alkaline phosphatase/ALP, urea/UREA, creatinine/CREA, Figure 4c). Importantly, histological analysis revealed no observable signs of inflammation or necrosis in multiple organs (heart, liver, spleen, lungs, and kidneys) from 101‐mer–treated mice (Figure 4d). Notably, even at high doses (50 µg per injection), 101‐mer was tolerated. Similarly, shorter arabinomannan motifs (18‐mer, 19‐mer, and 27‐mer) did not exhibit observable adverse effects (Figure S12). Collectively, these findings demonstrate that structurally defined arabinomannan oligosaccharides, particularly the 101‐mer, exhibit a favorable tolerability profile in vivo, supporting their further exploration as vaccine adjuvant candidates.

**FIGURE 4:**
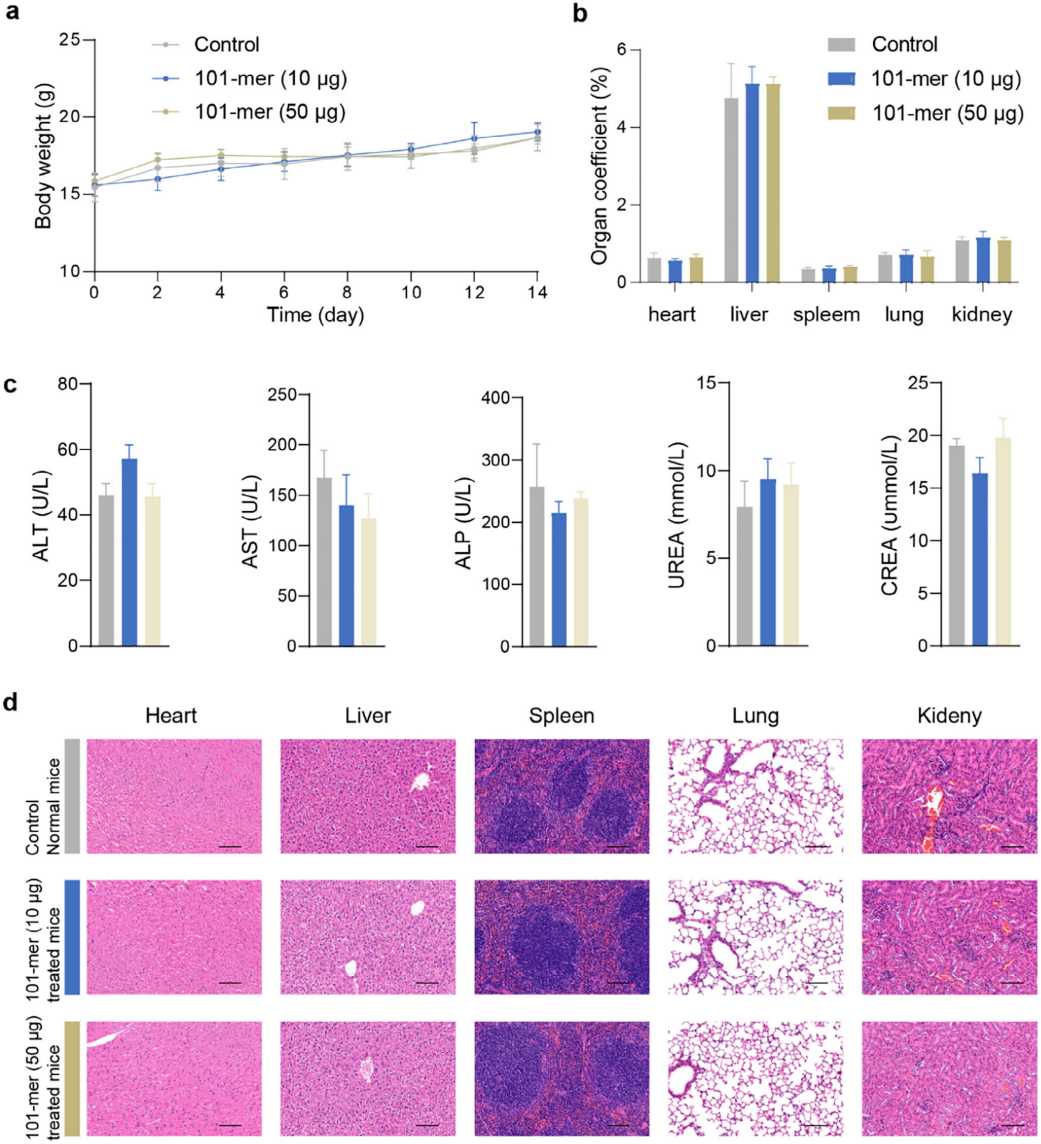
101‐mer exhibited a favorable tolerability profile in vivo. (a) Body weight of mice in control and 101‐mer groups (Control: with 100 µL PBS; 101‐mer: with 10–50 µg 101‐mer). Data represent the mean ± s.e.m. Statistical significance was tested with one‐way ANOVA. *n* = 5 mice per group. (b) Organ coefficient of mice in control and 101‐mer groups. Organ coefficient (%) = (organ weight/body weight) × 100%. (c) Serum biochemical parameters in control and 101‐mer groups, quantified using an automated animal blood biochemical analyzer (BS‐200, Mindray). (d) H&E staining of heart, spleen, and kidneys in control and 101‐mer groups on day 14. Scale bar, 100 µm.

This study demonstrates that synthetic oligosaccharides can overcome long‐standing hurdles in developing oligosaccharide‐based vaccine adjuvants. By constructing structurally defined arabinomannan motifs, we identified the 101‐mer as a potent immunostimulatory oligosaccharide that elicits robust immune responses while maintaining excellent biocompatibility. The translational potential of this synthetic platform is considerable. The 101‐mer not only enhanced protective immunity in influenza vaccination but also established a framework for extending oligosaccharide‐based adjuvants to other infectious diseases and cancer immunotherapy. More broadly, synthetic glycans represent a promising frontier in precision vaccinology, enabling rational design of next‐generation adjuvants with tunable potency and safety.

Nevertheless, challenges remain. While transcriptomic and docking analyses implicated the involvement of Dectin‐2 signaling, these findings should be interpreted as suggestive rather than definitive evidence of direct receptor engagement. Atomic‐resolution studies such as crystallography or cryo‐EM will be required to precisely define the minimal structural determinants of receptor engagement. Broader evaluation in diverse vaccine and therapeutic settings will also be essential to validate the generalizability of the 101‐mer's effects.

## Conflicts of Interest

The authors declare no conflicts of interest.

## Supporting information




**Supporting File**: advs74226‐sup‐0001‐SuppMat.docx

## Data Availability

The data that support the findings of this study are available in the supplementary material of this article.
