# Supplementary material for: Identification of Mannose‐Capped‐Arabinomannan 101‐mer as a Potential Influenza Virus Vaccine Adjuvant
Source: Adv Sci (Weinh). 2026 Feb 3;13(21):e19843. doi: 10.1002/advs.202519843 (PMC13073231; doi:10.1002/advs.202519843)
Supplement: Supplementary file 1 — Supporting File: advs74226‐sup‐0001‐SuppMat.docx [file ADVS-13-e19843-s001.docx]

Supporting Information
©Wiley-VCH 2021
69451 Weinheim, Germany

Identification of Mannose-Capped Arabinomannan 101-mer as A Potential Influenza Virus Vaccine Adjuvant

Yu-Fang Zhang,^#^ Ruihong Xu,^#^ Jia Luo, Yuxin Ma, Guozhi Xiao,* and Ye Liu*

**Abstract:** Many natural bacterial components as adjuvants can activate the host immune system, but the excessive toxicity and structure-identification challenge limit their applications and structure-activity relationship studies. Herein, we report the role of a series of chemically synthesized mannose-capped arabinomannan motifs from *Mycobacterium tuberculosis* cell wall, including 18-mer, 19-mer, 27-mer, and 101-mer in regulating host immunity. As an influenza vaccine adjuvant, 101-mer induced significantly enhanced anti-influenza antibody response and immune protection compared with other arabinomannan motifs. 101-mer elicited robust immunoenhancement while exhibiting a favorable tolerability profile, as it did not trigger any observable physiological toxicity or inflammatory reactions in various organs. Mechanistically, we found 101-mer may serve as a Dectin-2 agonist to activate host immunity through Syk/NF-κB signaling. This study provided a new oligosaccharide candidate which can satisfy the required ‘efficacy-safety’ balance for clinical adjuvant development.

DOI: 10.1002/anie.2021XXXXX

Table of Contents

1. Materials and Methods
2. Supplementary Figures
3. Author contributions

Materials and Methods

*Synthesis and characterization of arabinomannan motifs*

Arabinomannan motifs were synthesized and characterized following our established procedures.^[1]^

**18-mer:**

[α]_D_^25^ = +76.80 (C 0.30, H_2_O). ^1^H NMR (400 MHz, D_2_O) δ 5.26 (s, 1H), 5.22 – 5.18 (m, 3H), 5.18 – 5.14 (m, 3H), 5.12 (s, 1H), 5.11 – 5.07 (m, 6H), 5.06 (d, *J* = 1.9 Hz, 1H), 4.93 (d, *J* = 1.9 Hz, 3H), 4.84 (d, *J* = 1.8 Hz, 3H), 4.36 – 4.28 (m, 3H), 4.23 – 4.07 (m, 32H), 4.05 – 3.98 (m, 13H), 3.95 – 3.69 (m, 41H), 3.68 – 3.54 (m, 8H), 3.02 (t, *J* = 7.6 Hz, 2H), 1.73 – 1.64 (m, 4H), 1.51 – 1.42 (m, 2H). ^13^C NMR (151 MHz, D_2_O) δ 108.0, 107.6, 107.5, 107.43, 107.38, 107.35, 107.3, 105.6, 105.5, 105.4, 101.2, 100.7, 100.5, 100.4, 99.8, 87.5, 87.1, 87.0, 83.1, 83.0, 82.44, 82.41, 82.3, 82.24, 82.20, 81.7, 81.5, 81.2, 81.0, 80.8, 80.7, 80.6, 79.7, 79.5, 79.0, 76.8, 76.72, 76.68, 76.65, 76.6, 76.5, 75.93, 75.91, 75.03, 75.00, 74.96, 73.9, 72.8, 70.5, 69.9, 68.0, 67.93, 67.87, 67.83, 67.77, 66.8, 66.6, 66.4, 60.9, 60.6, 39.3, 28.0, 26.4, 22.2. MS (MALDI-TOF) calcd for C_98_H_163_NO_76_Na [M+Na]^+^ 2592.8813, found 2592.8811.

**19-mer:**

[α]_D_^25^ = +30.20 (C 0.10, H_2_O). ^1^H NMR (400 MHz, D_2_O) δ 5.18 (d, *J* = 1.2 Hz, 1H), 5.17 (d, *J* = 1.1 Hz, 1H), 5.15 (d, *J* = 3.1 Hz ,1H), 5.12 (s, 1H), 5.10 – 5.06 (m, 14H), 5.02 (s, 1H), 4.33 – 4.28 (m, 2H), 4.27 – 4.18 (m, 12H), 4.19 – 4.05 (m, 25H), 4.07 – 3.98 (m, 13H), 4.00 – 3.84 (m, 24H), 3.85 – 3.65 (m, 23H), 3.01 (t, *J* = 7.6 Hz, 2H), 1.74 – 1.64 (m, 4H), 1.52 – 1.43 (m, 2H). ^13^C NMR (151 MHz, D_2_O) δ 107.5, 107.43, 107.38, 107.36, 107.2, 107.1, 105.61, 100.57, 86.8, 83.9, 82.8, 82.4, 82.30, 82.26, 82.1, 82.0, 81.8, 81.6, 81.0, 80.9, 80.81, 80.75, 79.1, 76.7, 76.6, 76.52, 76.47, 76.4, 76.2, 74.8, 74.1, 68.0, 66.8, 66.7, 66.5, 66.4, 66.2, 62.9, 61.1, 60.5, 39.3, 28.0, 26.4, 22.2. MS (MALDI-TOF) calcd for C_100_H_165_NO_77_Na [M+Na]^+^ 2634.8919, found 2634.8919.

**27-mer:**

[α]_D_^25^ = +82.47 (C 0.105, H_2_O). ^1^H NMR (400 MHz, D_2_O) δ 5.16 (d, *J* = 1.6 Hz, 1H), 5.12 (s, 2H), 5.09 (d, *J* = 1.6 Hz, 23H), 5.03 (d, *J* = 2.1 Hz, 1H), 4.29 (s, 3H), 4.26 – 4.17 (m, 21H), 4.16 – 4.07 (m, 27H), 4.07 – 3.97 (m, 26H), 3.98 – 3.82 (m, 31H), 3.85 – 3.68 (m, 31H), 3.00 (t, *J* = 7.5 Hz, 2H), 1.72 – 1.60 (m, 4H), 1.50 – 1.40 (m, 2H). ^13^C NMR (151 MHz, D_2_O) δ 107.44, 107.40, 107.34, 107.25, 107.2, 107.0, 83.91, 83.85, 82.3, 82.1, 81.6, 81.2, 80.9, 80.8, 80.7, 79.0, 76.7, 76.48, 76.46, 76.3, 68.0, 66.8, 66.3, 66.2, 61.1, 61.0, 39.3, 28.0, 26.4, 22.2. MS (MALDI-TOF) calcd for C_140_H_229_NO_109_Na [M+Na]^+^ 3691.2299, found 3691.2295.

**101-mer:**

[α]_D_^25^ = +197.00 (C 0.10, H_2_O). ^1^H NMR (400 MHz, D_2_O) δ 5.25 (s, 2H), 5.21 – 5.13 (m, 21H), 5.12 (s, 6H), 5.11 – 5.04 (m, 65H), 5.02 (d, *J* = 1.8 Hz, 1H), 4.92 (s, 6H), 4.34 – 4.28 (m, 12H), 4.26 – 4.15 (m, 77H), 4.17 – 4.07 (m, 110H), 4.06 – 3.96 (m, 87H), 3.95 – 3.62 (m, 225H), 3.53 – 3.37 (m, 4H), 2.93 (t, *J* = 7.6 Hz, 2H), 1.66 – 1.58 (s, 4H), 1.54 – 1.38 (m, 2H). ^13^C NMR (201 MHz, D_2_O) δ 108.0, 107.64, 107.56, 107.49, 107.47, 107.38, 107.36, 107.2, 107.1, 105.62, 105.59, 105.5, 105.4, 101.1, 100.7, 100.6, 100.5, 100.4, 99.8, 87.5, 87.1, 87.0, 86.8, 83.11, 83.06, 83.0, 82.9, 82.8, 82.4, 82.31, 82.25, 82.2, 82.1, 82.0, 81.6, 81.3, 81.04, 80.98, 80.81, 80.76, 80.6, 79.72, 79.69, 79.1, 77.1, 76.8, 76.72, 76.68, 76.6, 76.53, 76.48, 76.41, 76.36, 76.2, 76.01, 75.95, 75.1, 75.03, 74.99, 74.8, 74.1, 73.9, 72.8, 70.5, 69.9, 68.0, 67.94, 67.88, 67.85, 66.82, 66.75, 66.7, 66.6, 66.4, 66.3, 66.2, 62.9, 60.9, 60.60, 60.58, 60.5, 39.3, 28.0, 26.4, 22.2. MS (MALDI-TOF) calcd for C_530_H_889_NO_411_ [M+Na]^+^ 13866.8587, found 13866.8532.

*Cells, mice, viruses, reagents*

DC 2.4 cells and MDCK cells were from American Type Tissue Collection (ATCC) and cultured in Dulbecco’s modified Eagle’s medium (DMEM, GIBCO) with 10% fetal bovine serum (v/v, FBS, GIBCO), 100 U/mL penicillin and 100 μg/mL streptomycin (GIBCO) at 37 °C in 5% CO_2_. C57BL/6 mice (6-8 weeks old) were purchased from the Institute of Medical Biology, Chinese Academy of Medical Sciences (CAMS), and housed in specific pathogen-free (SPF) condition at the Central Animal Care Services of Institute of Medical Biology, CAMS. Mice were randomly assigned into groups before each experiment. All mice experiments were conducted under protocols approved by the CAMS Animal Ethics Committee (approval number DWSP202203032). Influenza virus H1N1 was propagated in chicken eggs, and titrated in MDCK cells.

*Green fluorescent microspheres assay*

DC 2.4 cells were cultured and incubated with MF59 (5 μL per well), 18-mer (1 μg/mL), 19-mer (1 μg/mL), 27-mer (1 μg/mL), or 101-mer (1 μg/mL) for 12 h at 37 °C in 5% CO_2_, respectively. Green fluorescent microspheres (2 μm, 1 μL per well) were then added and incubated for another 6 h at room temperature (RT). Fluorescent intensity was quantified using a BioTek fluorescence analysis system (Biotek, cytation 1).

*Immunizations and influenza infection*

C57BL/6 mice (6-8 weeks old) were randomly divided into six groups: Ag group (10 μg HA per each injection), MF59 group (10 μg HA + 50 μL MF59), 18-mer group (10 μg HA + 10 μg 18-mer), 19-mer group (10 μg HA + 10 μg 19-mer), 27-mer group (10 μg HA + 10 μg 27-mer) and 101-mer group (10 μg HA + 10 μg 101-mer). Mice were immunized intramuscularly twice at two-week intervals. On day 24, blood samples were collected for ELISA. On day 28, mice were challenged intranasally (i.n.) with 10×LD_50_ H1N1. Following challenge, Body weight and mortality were monitored daily for 8 days. Mice exceeding a 25% loss of initial body weight were humanely euthanized. On day 8 post-infection, lung tissues were collected and fixed in 4% formaldehyde for H&E staining and lung injury scoring. ^[2]^

*Enzyme-linked immunosorbent assay (ELISA)*

HA-specific total IgG, IgG1, IgG2a, IgG2b, and IgG3 antibodies were quantified using ELISA. High-binding flat-bottom 96-well plate (Costar) was coated with HA protein (1 μg/mL) in carbonate coating buffer (Solarbio) overnight at 4 °C and blocked with PBS containing 2% BSA for 2 h at 37 °C. By washing the plate three times with PBST (PBS + 0.05% (v/v) Tween-20), sera from vaccinated mice were added with 1:2 serial dilutions in PBS plus 2% BSA at 37°C for 1 h. After washing the plate three times with, each well of 96-well plate was incubated with 100 μL HRP-conjugated anti-mouse IgG (1:40000, Invitrogen), IgG1 (1:10000, Invitrogen), IgG2a (1:1000, Invitrogen), IgG2b (1:1000, Invitrogen), and IgG3 (1:1000, Invitrogen) for 1 h at 37°C. Plates were washed again and developed with 3,3′,5,5′-tetramethylbenzidine (TMB, Solarbio, 100 μL) at RT for 10 min, then stopped with 2M H_2_SO_4_ (100 μL). The optical density (OD) was read at 450 nm and 630 nm wavelength by ELISA plate reader (Thermo Life Sciences), and endpoint titers were calculated with a cutoff three times the optical density over background.

*Hemagglutination Inhibiton (HAI) Assay*

HA-specific functional antibody titers were determined by HAI assay. Serial 2-fold dilutions of inactivated serum samples were incubated with an equal volume of four agglutinating doses of H1N1 at room temperature. Then, 1% chicken erythrocytes were added to each plate and incubated at room temperature for 30 min. HAI titers were defined as the reciprocal of the highest serum dilution that completely inhibited hemagglutination.

*Bulk transcriptional analysis*

Bulk RNA sequencing was performed on spleens from mice intramuscularly injected with 10 μg of the 101-mer (n = 3), with untreated mice serving as controls (n = 3). 3×10^6^ immune cells isolated from each spleen were lysed to harvest fresh RNAs for library construction and RNA sequencing. 150-bp paired-end reads were generated with the Illumina HiSeq platform. The clean RNA reads were mapped to the mouse reference genome (Ensembl build GRCm39) with STAR (version 2.7.5b; https://github.com/alexdobin/STAR). The transcriptional profiles analysis was performed with R package Deseq2. The biological process enrichment analysis was accomplished with BiNGO. The weighted gene coexpression network analysis (WGCNA) was conducted with R package WGCNA. The network was plotted with software CytoScape (version 3.9.1).

*Molecular docking analysis*

Molecular docking was performed using AutoDock Vina v1.1.2 to investigate interactions between four arabinomannan motifs (18-mer, 19-mer, 27-mer, and 101-mer) and murine Dectin-2 (UniProt: Q9JKF4). The receptor structure was predicted by AlphaFold (pTM = 0.71) and validated *via* structural superposition with human Dectin-2 (PDB: 8ROV), ensuring conservation of the calcium ion at the glycan-binding site.^[3]^ Protein preparation involved removal of water molecules, addition of polar hydrogens, and assignment of Kollman charges using AutoDock Tools. Carbohydrate ligands were first energy-minimized in ChemBio 3D using the MM2 Minimize method, followed by geometry optimization with the Forcite module at ultra-fine precision to obtain energetically favorable conformations.

*Immunohistochemical assay*

C57BL/6 mice (6-8 weeks old) were injected 101-mer (10 μg), 101-mer (50 μg), 18-mer (50 μg), 19-mer (50 μg), 27-mer (50 μg), and PBS (100 μL) into tail vein. The body weight of mice was weighed on days 0, 2, 4, 6, 8, 10, 12 and 14. On day 14, the heart, liver, spleen, lungs and kidneys in mice were harvested. The organ coefficient was calculated as the following formula: organ coefficient (%) = (organ weight/body weight) ×100%. The contention of AST, ALT, ALP, UREA and CREA in serum samples were detected by a fully automatic animal blood biochemical analyzer (BS-200, Mindray). The mouse organs (liver, kidney, spleen, heart and lungs) are harvested and use H&E staining to prepare pathological sections. Pathological sections are diagnosed and imaged using a photo-taking optical microscopy (Leica).

*Statistical analysis*

Data were presented as mean ± SD (standard deviation) or mean ± s.e.m. (standard error of mean) of triplicate determination. Statistical analysis was carried out using GraphPad Prism 9 Software. One-way analysis of variance (ANOVA) was used to test for statistical significance. When the p values were less than 0.05 (*P* < 0.05), differences were statistically significant.

References

[1] Y. Ma, Y. Zhang, Y. Huang, et al., "One-Pot Assembly of Mannose-Capped Lipoarabinomannan Motifs up to 101-Mer from the Mycobacterium tuberculosis Cell Wall*,*" *J. Am. Chem. Soc.* 146 (2024): 4112-4122.

[2] H. S. Kulkarni, J. S. Lee, J. A. Bastarache, et al., "Update on the Features and Measurements of Experimental Acute Lung Injury in Animals: An Official American Thoracic Society Workshop Report*,*" *Am. J. Respir. Cell Mol. Biol.* 66 (2022): e1-e14.

[3] H. Feinberg, S. A. F. Jegouzo, M. J. Rex, et al., "Mechanism of pathogen recognition by human dectin-2*,*" *J. Biol. Chem.* 292 (2017): 13402-13414.

Supplementary Figures


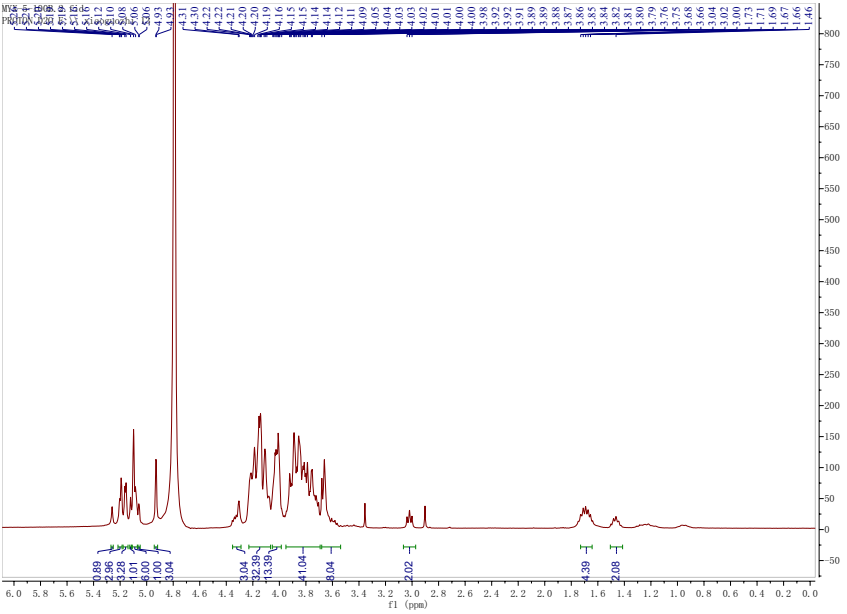


**Figure S1.** ^1^H NMR spectrum of 18-mer


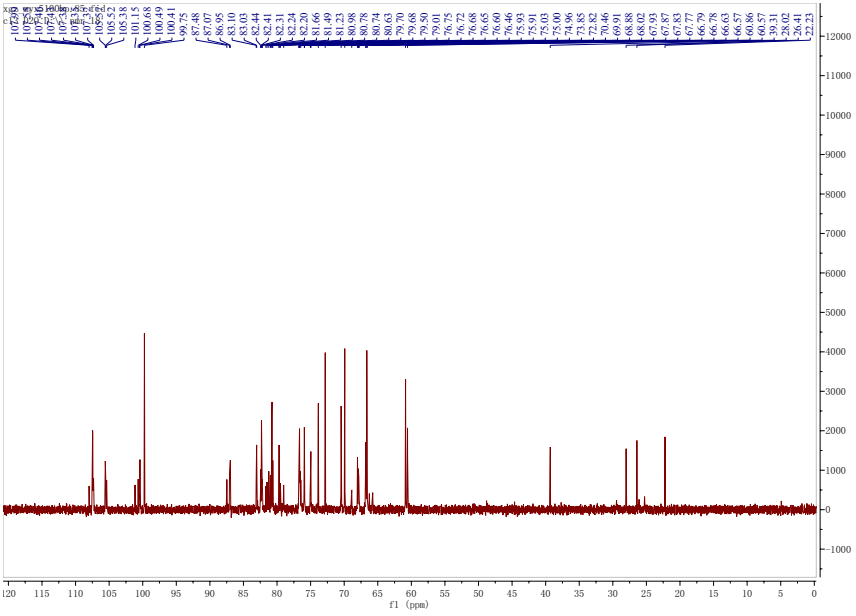


**Figure S2.** ^13^C NMR spectrum of 18-mer


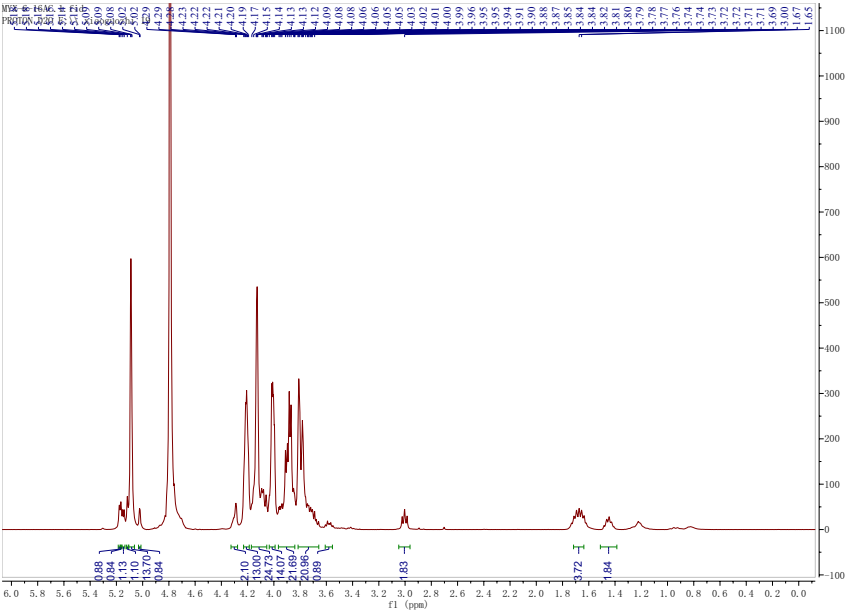


**Figure S3.** ^1^H NMR spectrum of 19-mer


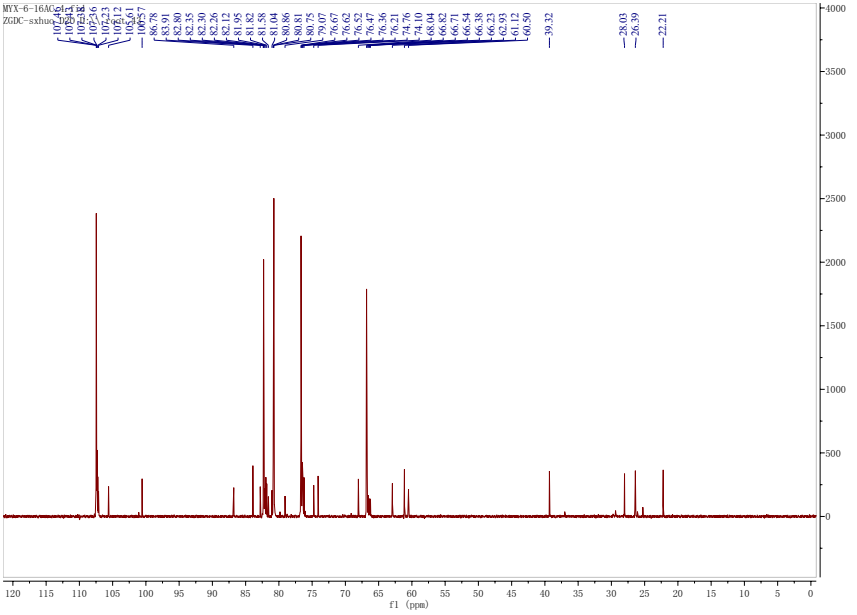


**Figure S4.** ^13^C NMR spectrum of 19-mer


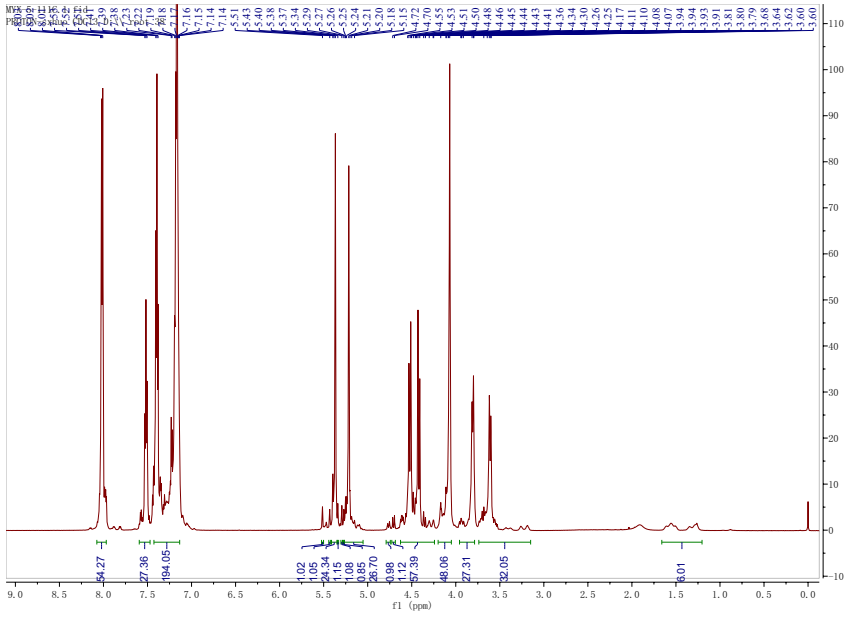


**Figure S5.** ^1^H NMR spectrum of 27-mer

**
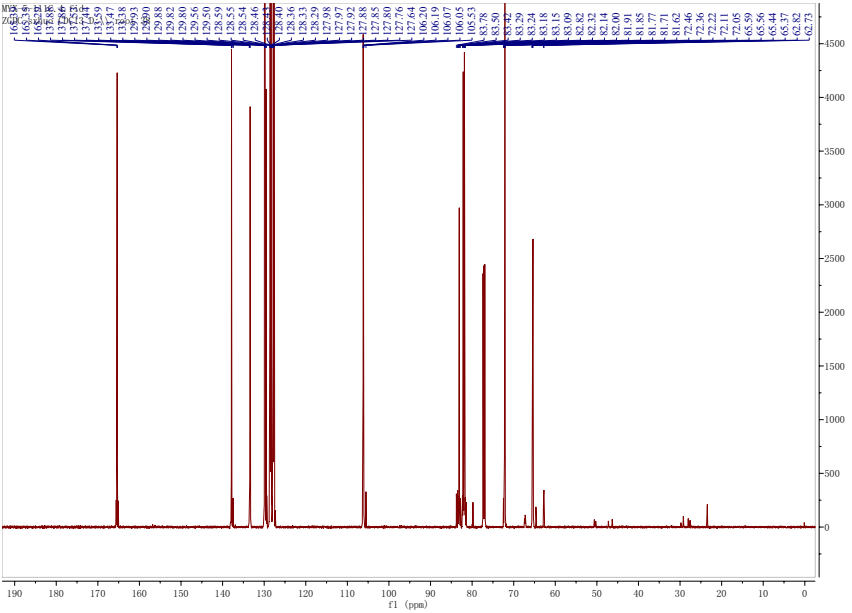
**

**Figure S6.** ^13^C NMR spectrum of 27-mer

**
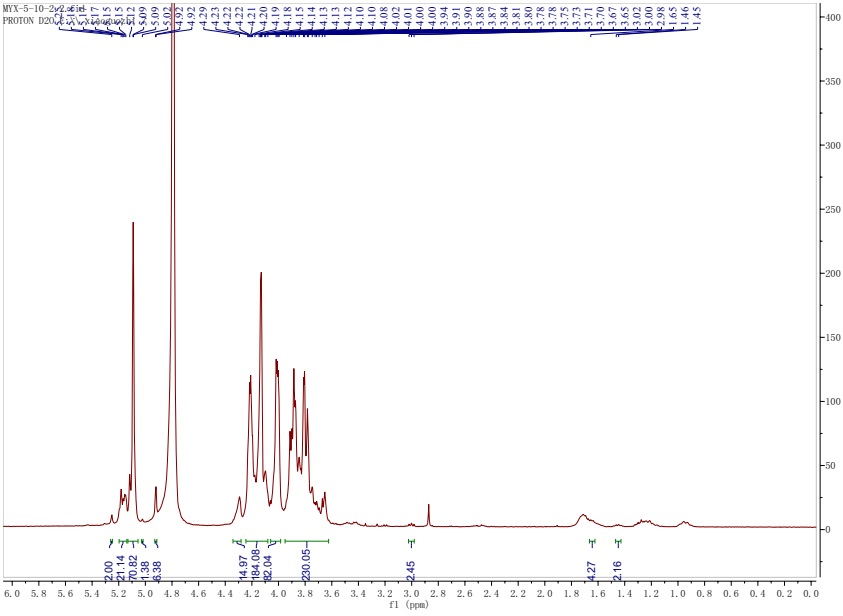
**

**Figure S7.** ^1^H NMR spectrum of 101-mer

**
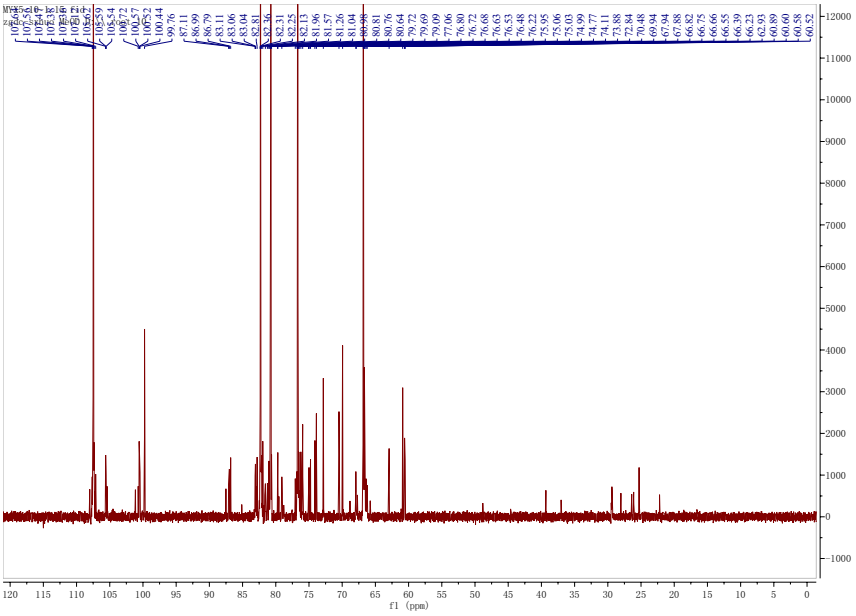
**

**Figure S8.** ^13^C NMR spectrum of 101-mer


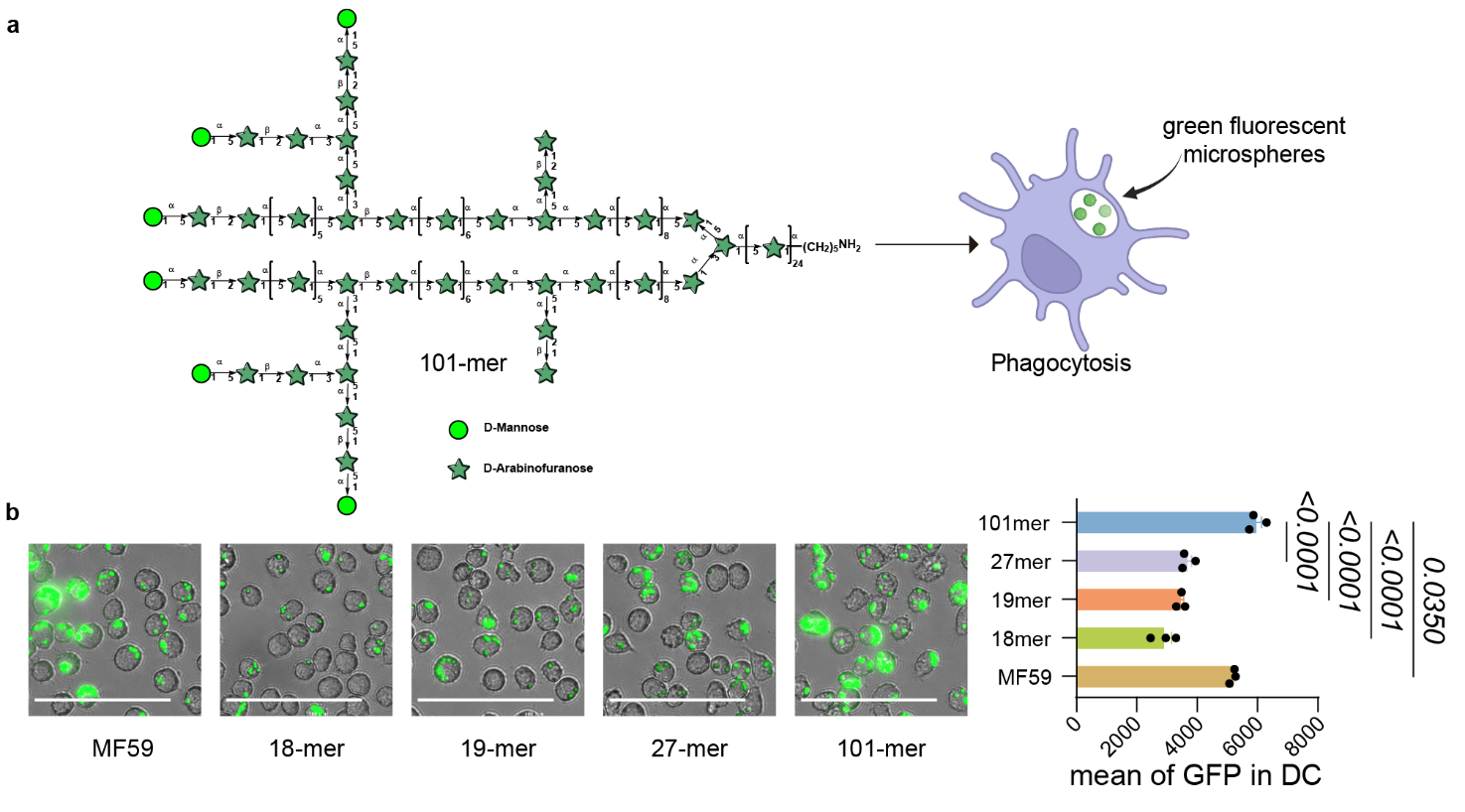


**Figure S9.** (a) Schematic illustration of enhanced DC phagocytosis by the arabinomannan 101-mer. (b) Representative fluorescence images and corresponding quantification of GFP intensity showing phagocytosis of four arabinomannan motifs and MF59 by DCs. Scale bar: 100 μm. All data were shown as mean ± s.e.m. The one-way ANOVA was used for statistical analysis. n = 3.


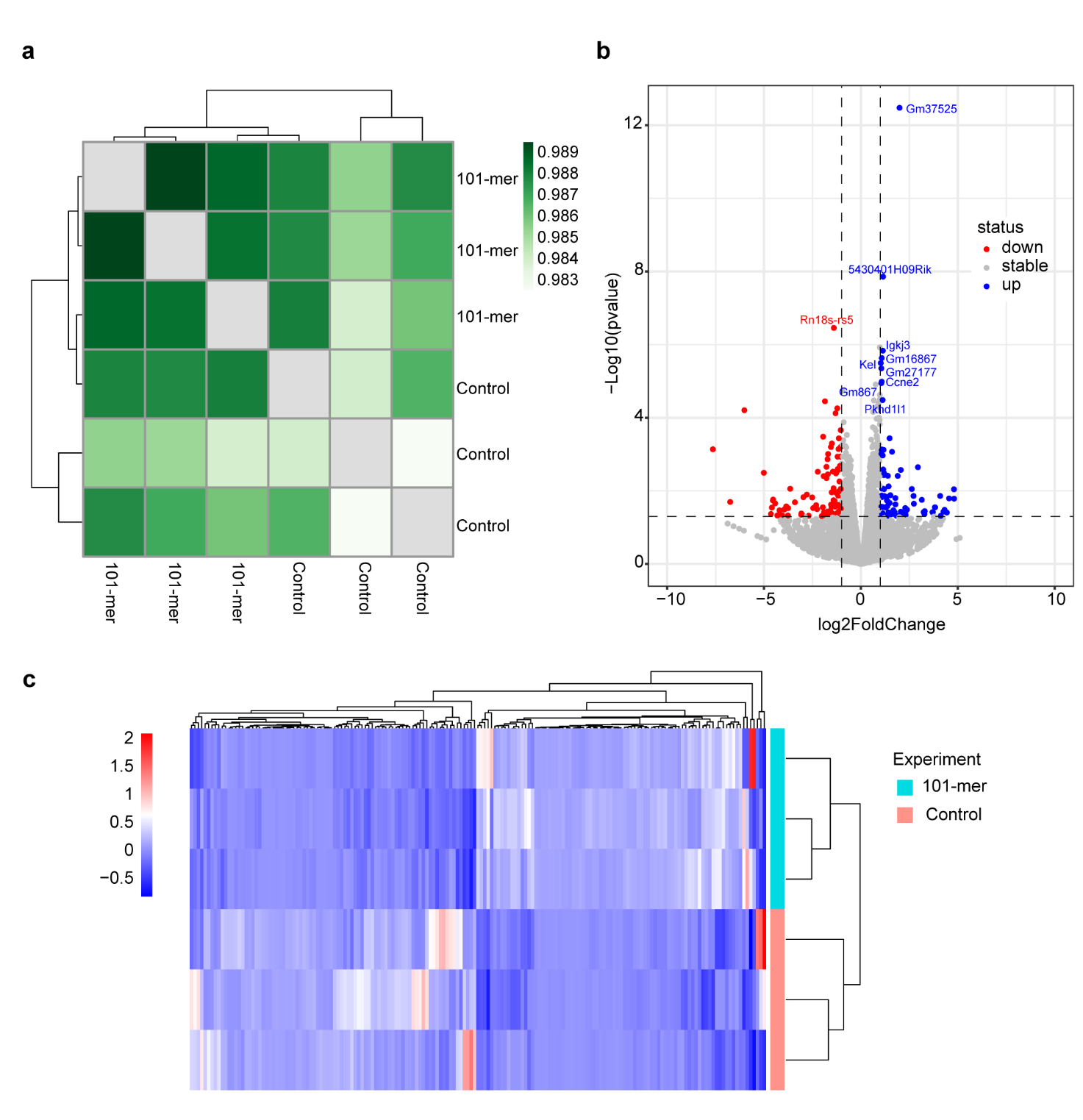


**Figure S10.** (a) Correlation heatmap of samples. (b) The volcano plot of DEGs. The horizontal gray line means *P*_adj_ = 0.01. The left vertical gray line means log2 fold change = -2. The right vertical gray line means log2 fold change = 2. (c) Heatmap of differentially expressed genes (DEGs).


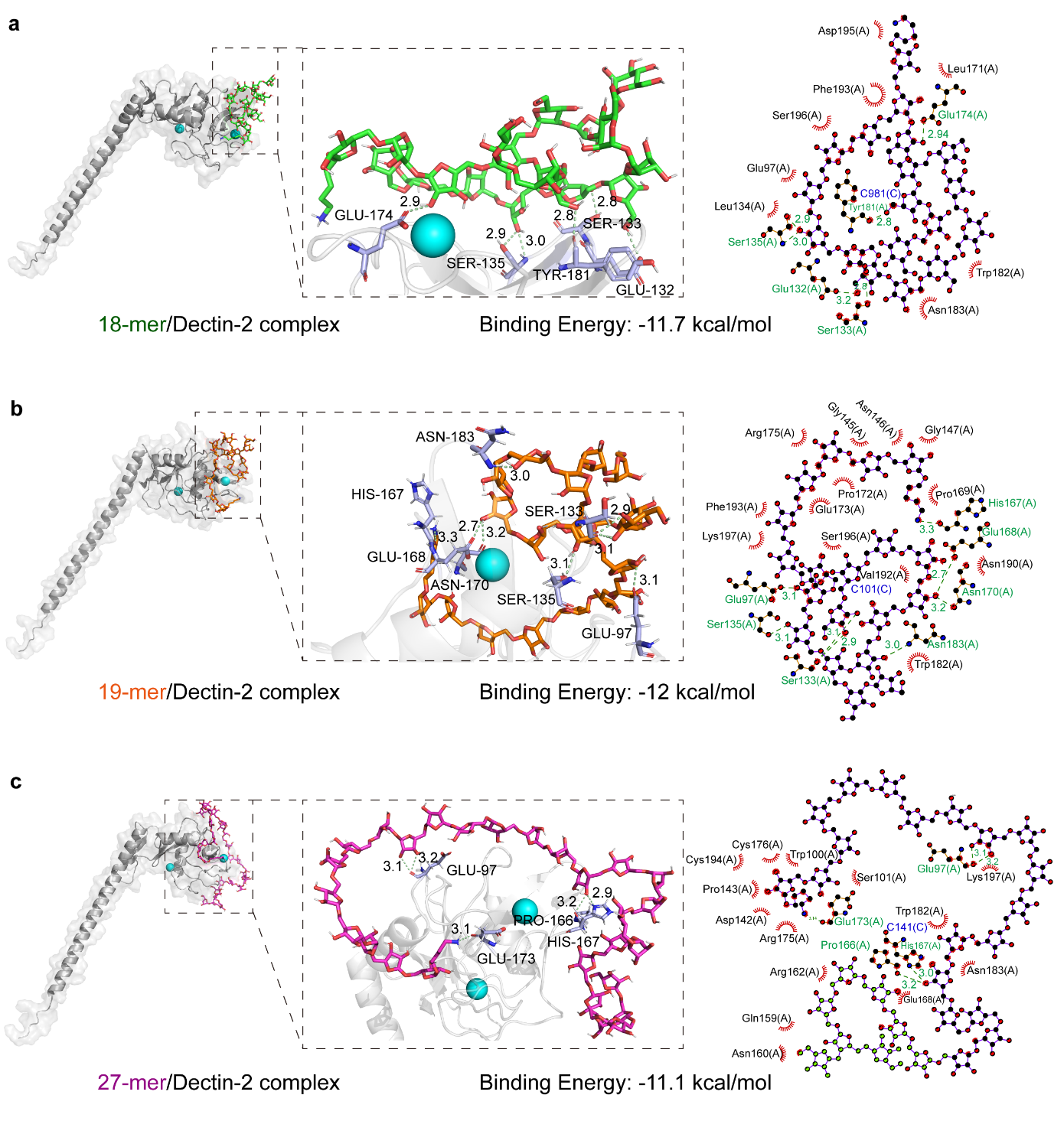


**Figure S11.** Molecular docking analysis of shorter arabinomannan motifs with Dectin-2. (a) Docking of the 18-mer (neon green) with the Dectin-2 CRD (gray) showed moderate affinity (–11.7 kcal/mol), primarily engaging SER135 and TYR181 (green). (b) Docking of the 19-mer (orange) revealed similar affinity (–12.0 kcal/mol), stabilized by an extended hydrogen-bond network with SER133, ASN183, and GLU168 (green). (c) Docking of the 27-mer (purple) yielded the weakest binding (–11.1 kcal/mol), mainly stabilized by interactions with GLU97 and GLU173 (green).


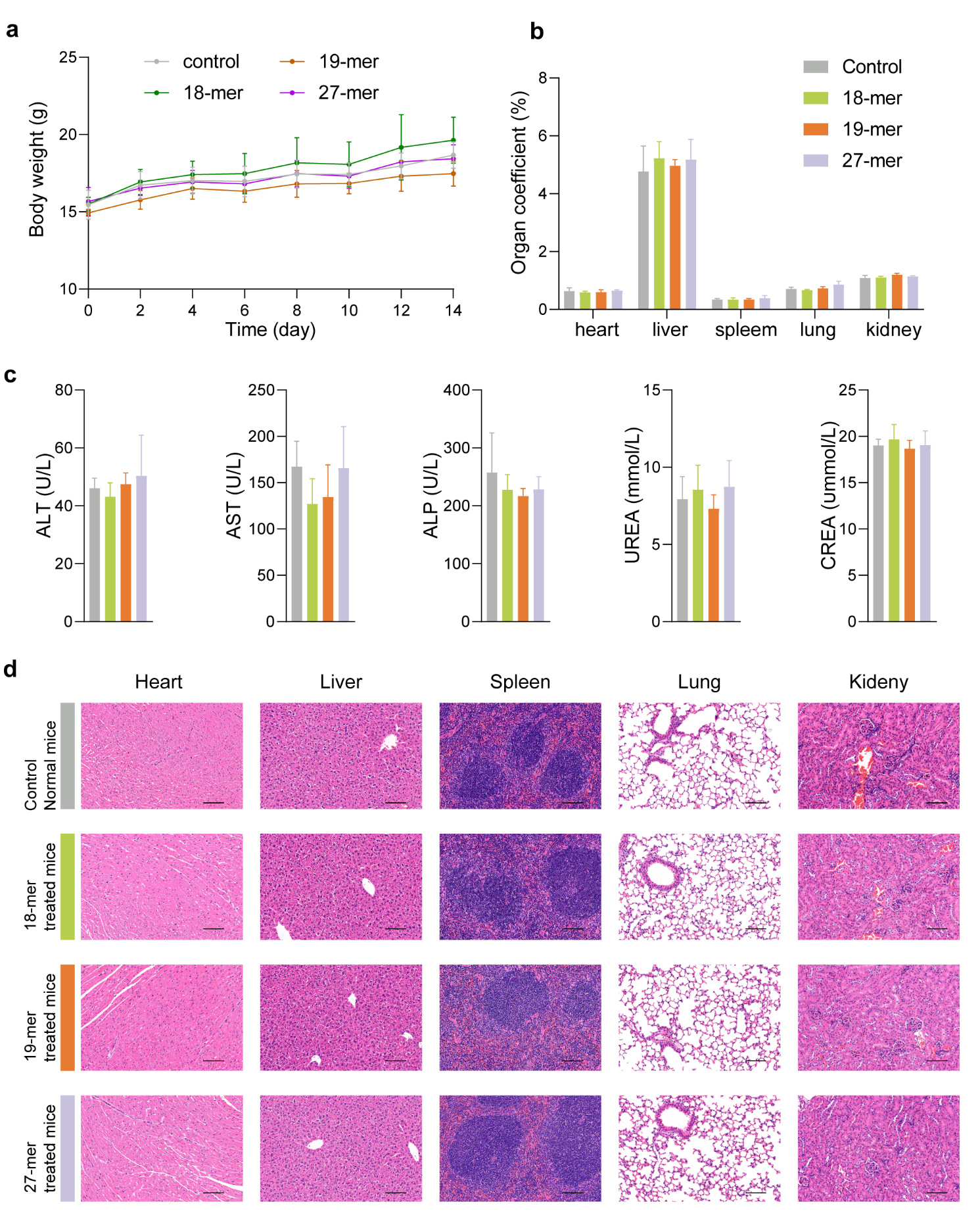


**Figure S12.** The biocompatibility evaluation of 18-mer, 19-mer and 27-mer. (a) Body weight of mice in control, 18-mer, 19-mer and 27-mer groups (Control: with 100 μL PBS; 18-mer, 19-mer or 27-mer groups: with 50 μg 18-mer, 19-mer or 27-mer). Data represents the mean ± s.e.m. Statistical significance was tested with one-way ANOVA. n = 5 mice per group. (b) Organ coefficient of mice in control, 18-mer, 19-mer and 27-mer groups. Organ coefficient (%) = (organ weight/body weight) ×100%. (c) Serum biochemical parameters in control, 18-mer, 19-mer and 27-mer groups. (d) Histological of heart, liver, spleen, lungs and kidneys in control, 18-mer, 19-mer and 27-mer groups. Scale bar = 100 μm.

# Author Contributions

Y.-F. Zhang designed the experiments, analyzed and interpreted data, and drafted the manuscript. Y.-F. Zhang, R. Xu and J. Luo performed the experiments *in vitro* and *in vivo*. Y. Ma and G. Xiao synthesized, analyzed and provided arabinomannan motifs. Y. Liu and G. Xiao led the project, interpreted the data and drafted the manuscript. All authors discussed the results and commented on the manuscript.
